# Tampon test measurement properties in women with provoked vestibulodynia: baseline data from a pragmatic randomized clinical trial

**DOI:** 10.1093/sexmed/qfag051

**Published:** 2026-06-29

**Authors:** Slawomir Wojniusz, Kristine Grimen Danielsen, Mette Bøymo Kaarbø, Anne Lise Ording Helgesen

**Affiliations:** Department of Rehabilitation Science and Health Technology, OsloMet - Oslo Metropolitan University, 0130 Oslo, Norway; Department of Rehabilitation Science and Health Technology, OsloMet - Oslo Metropolitan University, 0130 Oslo, Norway; Department of Pain Management and Research, Oslo University Hospital, 0424 Oslo, Norway; Norwegian Research Centre for Women’s Health, Oslo University Hospital, 0424 Oslo, Norway

**Keywords:** provoked vestibulodynia, tampon test, dyspareunia, pain measurement, vulvodynia

## Abstract

**Introduction:**

Provoked vestibulodynia (PVD) is a common cause of dyspareunia in young women. Because intercourse-related pain cannot be assessed consistently in all affected women, the tampon test (TT) is often used as a standardized proxy for insertional pain. This study aimed to evaluate within-person variability, measurement precision, and convergent validity of the TT, and to describe baseline sociodemographic and clinical characteristics of women enrolled in a pragmatic randomized clinical trial of PVD.

**Methods:**

Women aged 18-35 years with diagnosed PVD completed baseline questionnaires and 3 standardized TT assessments over 1 week. TT pain was rated on a 0-10 numerical rating scale (NRS). Baseline characteristics were described for the total sample and stratified by PVD onset type. Systematic change across TT repetitions, minimal detectable change at the 95% confidence level (MDC95), and correlations between TT pain and recalled pain during last intercourse were examined.

**Results:**

Eighty-six participants completed baseline assessment. Median age was 26.5 years, median symptom duration was 6.0 years, and 40% had primary PVD. Participants showed substantial symptom burden, including impaired sexual function, elevated psychological distress, and reduced health-related quality of life. Women with primary PVD reported higher TT pain than women with secondary PVD, whereas most other baseline characteristics were similar. Mean TT pain decreased slightly across repetitions from 5.1 to 4.6, although approximately 30% of participants showed fluctuations of ≥2 NRS points. Measurement precision improved when repeated TT scores were averaged: MDC95 decreased from 3.8 for a single TT to 2.7 for the mean of two tests and 2.2 for the mean of three tests. TT pain correlated moderately with pain during last intercourse (r = 0.46-0.49).

**Discussion:**

Women enrolled in this pragmatic PVD trial showed substantial symptom burden and clinical heterogeneity. Repeated TT assessments improved measurement precision and may therefore be preferable when insertional pain is used as an outcome in longitudinal PVD studies, particularly when evaluating within-person change or individual treatment response. TT pain showed partial convergent validity with recalled intercourse pain, but the 2 measures should not be considered interchangeable.

## Introduction

Provoked vestibulodynia (PVD) is a chronic vulvar pain condition characterized by pain localized to the vulvar vestibule and triggered by pressure, such as tampon insertion or attempted vaginal penetration.^1^ Prevalence estimates of 7%-16% have been reported, making PVD the most common subtype of vulvodynia and a major contributor to sexual pain disorders in young women.^2–5^ PVD can be further classified by onset. Primary PVD refers to pain present from the first attempts at vaginal penetration or tampon insertion, whereas secondary PVD refers to vestibular pain that develops after a period of pain-free penetration, tampon use, sexual activity, or regional touch.^6^ PVD is associated with marked interference in sexual functioning, relational problems, psychological distress, and reduced quality of life.^7^^,^^8^ Because PVD is a multifactorial condition without a clearly identifiable cause, patients’ own illness attributions may be clinically relevant. Understanding how women explain the origin of their symptoms may inform patient-centered communication, education, and individualized treatment planning.

Despite increasing research attention, PVD treatment studies remain difficult to compare because of heterogeneity in eligibility criteria and outcome definitions, and because insertional pain is inherently challenging to measure in a standardized manner.^9^^,^^10^ A major methodological challenge is that pain during intercourse cannot be measured consistently in all participants or at all time points. Many women with PVD avoid intercourse, report infrequent sexual activity, or are unable to engage in intercourse during the assessment period.^11^ To address these limitations, the tampon test (TT) has been proposed as a proxy for insertional pain that can be administered independent of sexual activity.^12^ TT has been recommended within current assessment frameworks for vulvar pain research,^9^^,^^13^ but uncertainties remain regarding optimal administration (eg, number of repetitions), stability of ratings, and its representativity for pain experienced during sexual activity.^14–16^

In a ProLoVe feasibility study,^14^ repeated TT ratings showed substantial within-person variability, supporting the need for repeated assessments and further evaluation in a larger sample. The present baseline report was designed primarily to evaluate measurement properties of the TT in women enrolled in the ProLoVe randomized clinical trial. Specifically, we examined short-term within-person variability in repeated TT ratings, quantified whether averaging repeated assessments improved measurement precision, and evaluated the association between TT pain and recalled pain during intercourse as an indicator of convergent validity. Baseline sociodemographic and clinical characteristics, including exploratory differences between primary and secondary PVD and participants’ own explanatory beliefs about symptom origin, are presented as clinical context for interpreting TT performance in a pragmatic and heterogeneous trial population.

## Methods

### Participants and recruitment

The ProLoVe trial was designed to reflect the heterogeneity of women with PVD typically encountered in clinical practice. Therefore, no restrictions were placed on relationship status, sexual activity, or prior treatment history. Participants were recruited through 3 pathways: (1) specialist referral from the Vulva Clinic at Oslo University Hospital, (2) referral from other gynecological specialists, and (3) responses to social media and online announcements. Eligible participants were women aged 18-35 years with PVD diagnosis recently confirmed by a gynecologist or dermatologist. Additional inclusion criteria were sufficient Norwegian language proficiency to complete questionnaires and ability to attend treatment in Oslo. Exclusion criteria were: (1) active infection in vulvar region; (2) dermatological genital lesions; (3) severe psychiatric conditions requiring specialist-level treatment; (4) vulvar pain not clearly provoked by intercourse, pressure on the vestibule, or tampon use.

### Baseline assessment

Baseline assessment included sociodemographic characteristics and standardized measures of pain, sexual function, psychological functioning, and health-related quality of life. Selection of outcome domains was informed by the Recommendations for the Study of Vulvar Pain in Women.^15^ The included outcome aligns closely with those later specified in the published core outcome set (COS) for treatment studies in PVD.^9^

Insertional nonsexual pain was assessed using TT.^12^ An unlubricated O.B.® ProComfort Normal tampon was inserted and immediately removed according to a standardized procedure, and maximum provoked vestibular pain was rated on a 0-10 numerical rating scale (NRS). Participants also recorded whether they were menstruating at the time of the test.^17^ TT assessments were completed on days 1, 3, and 5, and the mean score was used for analyses. Three TT assessments were used because the preceding ProLoVe feasibility study found substantial intra-individual variability across repeated TT ratings and concluded that repeated measurements are preferable when TT is used as an outcome.^14^ In the present study, three assessments were retained as a pragmatic compromise between improving measurement precision and limiting participant burden.

Insertional sexual pain was assessed retrospectively using recalled pain intensity during the most recent episode of vaginal intercourse (NRS 0-10) and the pain domain of the Female Sexual Function Index (FSFI).^18^

Sexual function was assessed using the FSFI. Scores are reported for the full study sample irrespective of recent sexual activity and separately for participants who had intercourse during the preceding 4 weeks.

Pain quality, pain-related interference, and pain coping were assessed using the Vulvar Pain Assessment Questionnaire.^19^ Pain self-efficacy was assessed using a single question “How confident are you that you can manage your vulvodynia pain on your own?” and rated on a 5-point verbal rating scale.

Psychological functioning was assessed using the Pain Catastrophizing Scale,^20^ the 10-item Ruminative Response Scale^21^ and the Hopkins Symptom Checklist (HSCL-25).^22^ Health-related quality of life was measured using EuroQol Health Scale (EQ-5D-5L) with Norwegian population based preference weights.^23^^,^^24^

Participants could also specify, through a free text response, their own explanations for the origin of their PVD. Responses indicating no answer were excluded. Free-text responses were analyzed exploratively using a pragmatic thematic descriptive content approach. One researcher read all responses repeatedly in the original language and generated initial codes inductively from the data. Because many participants described several possible explanations, individual responses could be assigned to more than one code or theme. Codes were then grouped into broader descriptive themes reflecting recurring patterns in participants’ perceived symptom attributions. Although the coding was inductive, interpretation was informed by a broad biopsychosocial understanding of PVD. The analysis was intended to contextualize baseline characteristics rather than to constitute a full qualitative substudy.

The ongoing RCT was registered with prespecified longitudinal treatment outcomes, including TT as a repeated secondary outcome summarized as the mean of 3 measurements at each time point (ClinicalTrials registration NCT04613713). The present analyses of TT measurement properties at baseline, including within-person variability, measurement precision, and convergent validity, were planned for this baseline report based on findings from the earlier mixed-methods feasibility study, but were not specified in the public trial registry.

### Statistical analysis

Statistical analyses were performed using IBM SPSS Statistics for Windows, version 31.0 (IBM Corp., Armonk, NY, USA) and the R version 4.5.2 (R Foundation for Statistical Computing, Vienna, Austria) was used to generate graphical spaghetti plot. All tests were 2-sided, and *P* < .05 was considered statistically significant. Analyses were conducted using available data. Because one participant was missing the third TT assessment, analyses requiring complete data across all three TT repetitions, including the repeated-measures GLM, ICC, SEM, and MDC95 calculations, were based on 85 complete cases. Because only 1 TT value was missing, no imputation of missing data or formal sensitivity analysis was performed.

Descriptive statistics are presented as medians with interquartile ranges (IQR) for continuous variables and as frequencies and percentages for categorical variables. Comparisons between women with primary and secondary PVD were conducted as exploratory baseline subgroup analyses. No formal adjustment for multiple testing was applied; therefore, *P*-values from these comparisons should be interpreted as nominal and hypothesis-generating.

A repeated-measures general linear model (GLM) was used to assess whether mean TT pain changed systematically across the three baseline repetitions. Sphericity was assessed using Mauchly’s test. Because Mauchly’s test was non-significant, sphericity-assumed results are reported. When a significant overall time effect was detected, pairwise comparisons with Sidak correction were applied. Individual TT trajectories were visualized using a spaghetti plot. The association between menstruation status (yes/no) and TT pain was examined using a linear mixed model including the three baseline TT repetitions. Menstruation status and repetition were included as fixed effects, with a random intercept for participant to account for repeated measurements. A mixed model was chosen here because menstruation status could differ across the three test occasions within the same participant.

Measurement precision of TT was evaluated using the minimal detectable change at the 95% confidence level (MDC95). Test–retest reliability was estimated using an intraclass correlation coefficient (ICC) from a 2-way mixed-effects, absolute-agreement model. The standard error of measurement (SEM) was calculated as $SEM= SD\times \sqrt{1}- ICC$, where SD reflected the observed variability of the repeated baseline TT assessments. MDC95 for a single TT score was calculated as $1.96\times \surd 2\times SEM$. For the mean of k repeated TT scores, SEM was divided by $\surd k$, and MDC95 was calculated as $1.96\times \sqrt{2}\times \left(\frac{SEM}{\sqrt{k}}\right)$, where *k* = 2 or 3. The 95% CI for the ICC was obtained from the reliability model. Confidence intervals for SEM and MDC95 were derived from the ICC confidence limits while treating the observed SD as fixed; these intervals should therefore be interpreted as approximate.

Convergent validity was assessed using Pearson’s correlation coefficients (*r*) between TT pain scores and pain intensity score during the last intercourse.

### Data collection and ethics

The study was approved by the Regional Committees for Medical and Health Research Ethics (REC South East; 2019/1290) and registered at ClinicalTrials.gov (NCT04613713). The project was approved and registered at Oslo Metropolitan University in accordance with institutional procedures (Id: 10161) and the processing and management of personal data were approved by Sikt – Norwegian Agency for Shared Services in Education and Research (SIKT protocol nr. 652720). Before enrollment, participants provided written informed consent. All data were self-administered electronically using Nettskjema (nettskjema.no) and stored securely on the Services for Sensitive Data (TSD) platform. Participants completed the questionnaires at their convenience using a computer or smartphone. Data are collected at baseline and at 6- and 12-month follow up. The present report includes baseline data only.

## Results

Baseline characteristics are presented first to describe the pragmatic trial population in which TT measurement properties were evaluated. The main measurement-property analyses are then reported in relation to within-person stability, measurement precision, and convergent validity.

Baseline sociodemographic and clinical characteristics for the total sample, and for the subgroups of primary and secondary PVD, are presented in Tables 1 and 2. The sample consisted predominantly of young women who were employed or studying and who reported long standing PVD symptoms associated with substantial pain-related sexual impairment. Participants showed considerable heterogeneity in provoked pain intensity and sexual activity patterns, including a subgroup of women with primary PVD who had never engaged in intercourse. Avoidance oriented coping, elevated psychological distress, and reduced health-related quality of life were frequently observed.

**Table 1:** Sociodemographic characteristics.

Sociodemographic characteristics	All (*n* = 86)	Primary PVD (*n* = 34)	Secondary PVD (*n* = 52)
**Age (years)**	26.5 (23-30)	25.5 (23-30)	27.0 (23-30)
**BMI (kg/m** ^**2**^ **)**	22.8 (21-25)	22.8 (20-26)	22.8 (21-24)
**Highest completed education**			
Primary education	3 (9)	2 (6)	1 (2)
Upper secondary education	22 (26)	12 (35)	10 (20)
Undergraduate education	31 (36)	10 (29)	21 (40)
Postgraduate education	30 (35)	10 (29)	20 (39)
**Working status**			
Student	33 (38)	13 (38)	20 (38)
Full-time employed	41 (48)	15 (44)	26 (50)
Part-time employed	27 (31)	8 (24)	19 (37)
Fully on sick leave	4 (5)	2 (6)	2 (4)
Partially on sick leave	6 (7)	2 (6)	4 (8)
Disability pension	1 (1)	1 (3)	0
**Working ability (0-10)**	7.8 (6-9)	7.5 (6-10)	7.8 (6-9)
**Physically active (PA index)**	62 (72)	22 (65)	40 (77)
**Relationship Status**			
Married/cohabiting	39 (45)	12 (35)	27 (52)
In a relationship	20 (23)	7 (21)	13 (25)
Single	27 (31)	15 (44)	12 (23)
**Biological children**	5 (6)	1 (3)	5 (10)

Abbreviation: PVD = provoked vestibulodynia. Data are presenter as median (Q1-Q3) or as *n* (%). Physical activity (PA index) reflects participants meeting the predefined criterion for being physically active according to the Physical Activity index.^25^^,^^26^

**Table 2:** Clinical characteristics.

Clinical characteristics	All (*n* = 86)	Primary PVD (*n* = 34)	Secondary PVD (*n* = 52)
**Pain duration (years)**	6.0 (3-10)	10 (5-12)	5 (3-7)
**Last intercourse**			
<1 month	39 (45)	13 (38)	26 (50)
1–3 months	20 (23)	6 (18)	14 (27)
6–12 months	11 (13)	6 (18)	5 (10)
>12 months	13 (15)	6 (18)	7 (14)
Never had intercourse	3 (4)	3 (9)	0
**Pain during last intercourse (0-10) *n* = 83**	6 (4-8)	7 (5-8)^a^	6 (4-6)
Mild (2-3)	14 (17)	4 (13)^a^	10 (19)
Moderate (4-6)	36 (43)	11 (35)^a^	25 (48)
Severe (7-10)	33 (40)	16 (52)^a^	17 (33)
**Tampon test pain (0-10), average of 3 tests**	4.2 (3-6)	5.3 (3-9)	3.8 (3-5)
Mild (1-3)	31 (36)	10 (29)	26 (50)
Moderate (4-6)	38 (44)	9 (26)	15 (29)
Severe (7-10)	17 (20)	15 (44)	11 (21)
**FSFI total (*n* = 86; intercourse last 4 weeks, *n* = 51)**	18.0 (13-22); 20.6 (16-23)	17.3 (11-21)	18.9 (13-23)
FSFI desire	3.0 (2.3–3.6); 3,0 (2.4-3.6)	3.0 (1.8-3.6)	3.0 (2.4-3.6)
FSFI arousal	3.3 (1.8-4.2); 3.3 (2.4-4.2)	3.3 (1.4-4.2)	2.9 (1.9-4.2)
FSFI lubrication	3.3 (1.8-5.1); 3.3 (2.4-5.4)	3.8 (1.4-5.5)	3.2 (2.1-5.0)
FSFI orgasm	4.2 (2.4-5.2); 4.4 (3.2-5.6)	4.0 (0.9-5.2)	4.4 (2.9-5.0)
FSFI satisfaction	3.2 (1.2-4.4); 3.6 (2.8-4.4)	2.0 (1.1-4.4)	3.2 (1.6-4.3)
FSFI pain	1.2 (0.0-2.0); 2.0 (1.2-2.8)	0 (0.0-2.0)	1.2 (0.0-2.3)
**VPAQ—impact on daily activities (a lot/very much)**			
Wearing tight-fitting clothing	30 (35)	11 (32)	19 (37)
Activities involving pressure	24 (18)	8 (24)	16 (31)
Sitting	15 (17)	8 (24)	7 (13)
**VPAQ—coping Strategies (often/always)**			
Avoid anything that might cause pain	58 (67)	27 (79)	31 (60)
Breathe deeply	38 (44)	15 (44)	23 (44)
Relax my body	28 (32)	14 (41)	14 (27)
**VPAQ—pain descriptors**			
Burning pain	4.0 (3-5)	4.0 (4-5)	4.0 (3-5)
Incisive pain	3.5 (3-4)	3.8 (3-5)	3.0 (2-4)
Sensitivity	3.5 (3-4)	3.5 (3-4)	3.5 (3-4)
**PVD-related self-efficacy (VRS 1-5)**	4.0 (3-4)	4.0 (4-5)	4.0 (3-4)
**PVD pain catastrophizing (0-52)**	29.0 (24-36)	28.5 (23-37)	30.5 (24-35)
**HSCL-25 total (1-4)**	2.2 (1.8–2.7)	2.1 (1.7-2.4)	2.2 (1.7-2.7)
HSCL—depression (1-4)	2.2 (1.9–2.7)	2.2 (1.8-2.7)	2.2 (1.8-2.8)
HSCL—anxiety (1-4)	2.0 (1.6–2.3)	1.9 (1.5-2.2)	2.1 (1.6-2.5)
**RRS-10 (0-40)**	23.5 (20-28)	23.5 (20-29)	23.5 (20-28)
**EQ-5D-5L health scale (0–100)**	67.5 (50-80)	70.0 (59-80)	65.0 (50-79)
**EQ-5D-5L index**	0.78 (0.63-0.87)	0.78 (0.61-0.87)	0.76 (0.65-0.88)

^a^ *n* = 31 (participants who had never engaged in intercourse were excluded from the analysis). Abbreviations: EQ-5D-5L = EuroQol Health Scale; FSFI = Female Sexual Function Index; HSCL = Hopkins Symptom Checklist; PVD = provoked vestibulodynia; RRS = Ruminative Response Scale; VPAQ = Vulvar Pain Assessment Questionnaire; VRS = Verbal Rating Scale. FSFI domain and total scores are reported for the full sample (*n* = 86) and separately for participants reporting intercourse during the past 4 weeks (*n* = 51). VPAQ items are reported as the proportion endorsing “a lot” or “very much” for impact items and “often” or “always” for coping strategies. Data are presenter as median (Q1-Q3) or as *n* (%)*.*

Comorbid conditions were reported by 49% of participants, most commonly musculoskeletal pain conditions and psychological disorders. Of the 86 participants, 58% had previously received conservative treatment for PVD, predominantly physiotherapy and/or osteopathy, while 45% had received medical treatment, most commonly lidocaine based topical agents. At baseline, 21% of participants reported daily use of pain medication related to PVD.

Free-text responses suggested that participants most frequently attributed their symptoms to physical or biological factors, often in combination with other explanations. Commonly reported attributions included recurrent yeast infections, urinary tract infections, endometriosis, and pelvic floor muscle tension. Many participants also described psychological or emotional contributors, particularly stress and anxiety, while others referred to sexual and relational experiences, including painful sexual debut, negative sexual experiences, and intercourse despite pain. Nine women reported sexual abuse or trauma as a possible contributing factor.

### Primary vs. secondary PVD

Exploratory, subgroup comparisons suggested that a higher proportion of women with secondary PVD were in a romantic relationship compared with women with primary PVD (Chi-square test, nominal *P* = .038). Women with primary PVD reported higher TT pain scores than women with secondary PVD (Mann–Whitney *U* test, nominal *P* = .008). Notably, 6 of 34 women with primary PVD (18%) reported a maximal TT pain score of 10 on all three repetitions, whereas none in the secondary PVD group did so. No formal adjustment for multiple testing was applied, and these subgroup findings should therefore be interpreted cautiously.

### Tampon test performance

#### Within person stability

**Figure 1:**
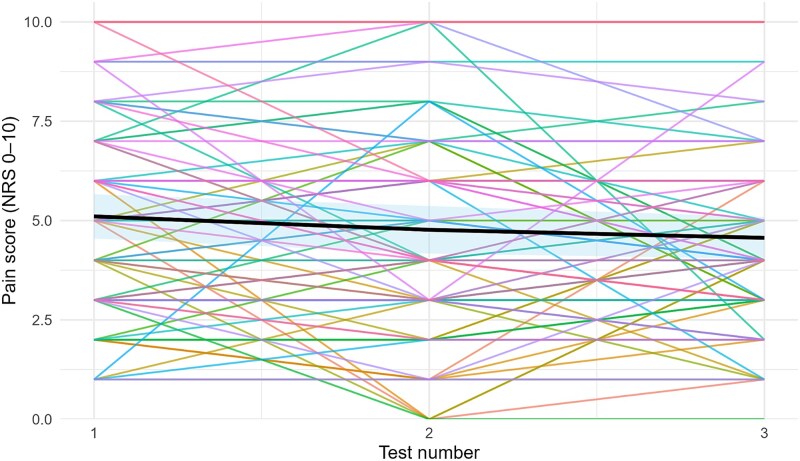
Individual trajectories in tampon test pain scores. Each colored line represents a participant’s repeated pain ratings. The thick black line shows the mean rating at each test repetition, with the shaded band indicating its 95% CI.

Individual trajectories of the three TT pain ratings are presented in Figure 1. Mean TT pain decreased slightly across the three test days, from 5.1 to 4.8 and 4.6, yielding a significant overall time effect (F(2,83) = 3.82; *P* = .026). Only the comparison between the first and third test reached statistical significance (mean difference = 0.5; *P* = .028). TT pain did not differ by menstruation status (mean difference = −0.4; *P* = .27).

Visual inspection of individual trajectories revealed substantial within-person variability, with crossing lines and heterogeneous patterns across repeated measurements. Approximately 30% of participants exhibited pain fluctuations of two or more NRS points between test repetitions (Figure 1).

#### Measurement precision (MDC95)

In the 85 participants with complete TT data across all three baseline assessments, the single-measure absolute-agreement ICC was 0.73, 95% CI, 0.64-0.80. The SEM was 1.38, with a 95% CI, 1.19-1.59 derived from the ICC confidence limits, and the corresponding MDC95 for a single TT was 3.8 NRS points, with a 95% CI, 3.3-4.4. For the mean of 2 TT repetitions, MDC95 was 2.7 NRS points, 95% CI, 2.3-3.1, and for the mean of 3 TT assessments, MDC95 decreased to 2.2 NRS points, 95% CI, 1.9-2.6.

#### Association between TT pain and pain during last intercourse

Three participants who had never engaged in intercourse were excluded from this analysis. Pearson correlation coefficients between TT pain and recalled pain during last intercourse were moderate, ranging from 0.46 for individual TT measurements to 0.49 for the mean of three TT measurements (all *P* < .001). We also conducted an exploratory analysis stratified by time since last intercourse, which varied considerably across participants (Table 2). These analyses yielded unstable and inconsistent correlation estimates, likely reflecting small subgroup sizes and variable recall periods. The subgroup correlations were therefore not interpreted further. Overall, the association between TT pain and intercourse-related pain was moderate and was not meaningfully affected by whether TT pain was represented by an individual measurement or by the mean of repeated measurements.

## Discussion

The main contribution of this study is the empirical evaluation of short-term TT measurement properties in a pragmatic sample of women with PVD. The findings show substantial within-person variability in repeated TT pain ratings, demonstrate that averaging repeated TT assessments improves measurement precision, and support partial convergent validity between TT pain and recalled intercourse-related pain. The baseline characterization provides important clinical context for these findings, showing that TT performance was evaluated in a heterogeneous trial population with long-standing symptoms, substantial sexual impairment, elevated psychological distress, and varied sexual activity patterns.

### Clinical context: baseline characteristics

In line with other clinical PVD studies,^27–29^ participants were predominantly highly educated women in their mid-twenties. Educational attainment exceeded that of the general Norwegian female population in the same age group^30^ but was closer to levels reported in urban Norwegian areas.^31^ Nevertheless, the relatively high educational level of the sample should be considered when interpreting the generalizability of the findings.

More than 70% of participants were classified as physically active according to the Physical Activity Index,^25^ a proportion similar to that reported in the general Norwegian female population.^26^

Psychological distress, measured by the HSCL-25, was substantially higher than Norwegian normative values.^32^ Participants also reported elevated levels of PVD-related pain catastrophizing and rumination.^20^^,^^21^ Health-related quality of life, assessed using the EQ-5D-5L index and VAS, was reduced compared with Norwegian age- and sex-specific norms.^24^ These psychological factors may influence pain perception and are therefore relevant when interpreting TT variability. Pain perception in PVD is influenced by biopsychosocial factors, and anxiety, stress, pain catastrophizing, pain-related fear, hypervigilance, and low pain acceptance may shape both anticipated and experienced pain during provocation.^8^ These factors could contribute to day-to-day variation in TT scores, although the present study did not examine psychological variables as moderators of TT pain. The free-text responses further support the clinical relevance of a biopsychosocial approach: participants often described symptom onset as multifactorial, combining biomedical, muscular, emotional, sexual, relational, and trauma-related explanations. These illness attributions may be important to address in clinical encounters, as they can influence expectations, coping strategies, and openness to different treatment components.

Pain intensity during intercourse was approximately one NRS point lower than that reported in recent randomized trials.^27^^,^^29^ This difference is likely attributable to the pragmatic design of the present study, which did not impose eligibility requirements related to recent sexual activity, relationship status, or minimum pain severity. The proportion of women reporting symptom onset from their first attempt at intercourse or tampon use was comparable to that observed in other clinical PVD studies.^28^^,^^29^

Sexual function, assessed with the FSFI, was markedly impaired according to the commonly used cut off score of 26.55,^33^ both in the full sample and among women who had intercourse during the preceding four weeks. FSFI scores were similar to those reported in other PVD populations.^27^^,^^29^^,^^34^

When stratified by onset type, women with primary PVD reported higher provoked pain intensity on the TT than women with secondary PVD, whereas most other baseline clinical characteristics were similar across the groups. In addition, a higher proportion of women with secondary PVD were in a romantic relationship. The higher TT pain observed among women with primary PVD is clinically interesting and is consistent with the possibility that primary PVD may be associated with greater provoked insertional pain. However, these subgroup comparisons were exploratory, based on modest subgroup sizes, and not adjusted for multiple comparisons. They should therefore be interpreted cautiously and require confirmation in larger studies with prespecified subgroup analyses.

Overall, the demographic and clinical profile of the sample characterized by high educational attainment, elevated psychological distress, pain catastrophizing, impaired sexual function, and reduced health-related quality of life is broadly consistent with previous PVD studies. Despite this burden, participants demonstrated relatively high levels of self-efficacy and work ability. This may partly reflect prior exposure to conservative and medical treatments for PVD, reported by the majority of the sample, as well as the fact that pain is most frequently provoked in a sexual context.

### Within person variability in tampon test pain ratings

A key finding of this study is the pronounced within person variability in TT pain ratings across three closely spaced administrations. Although a small mean reduction in pain was observed across test repetitions, visual inspection of individual trajectories revealed heterogeneous patterns characterized by frequent crossing lines and clinically relevant fluctuations (Figure 1). Approximately one third of participants exhibited changes of two or more NRS points between repetitions, exceeding commonly used thresholds for clinically important change in pain outcomes.^35^

These findings extend observations from the ProLoVe feasibility study^14^ and align with evidence from other chronic pain conditions, in which short term pain fluctuations are common even in the absence of intervention.^36–38^ The observed variability is unlikely to be explained solely by learning or habituation effects and instead suggests that provoked vestibular pain is inherently unstable at the individual level. This has important implications for outcome assessment in PVD trials that rely on single occasion pain measurements.

### Measurement precision and implications for trial design

Analysis of the minimal detectable change (MDC95) demonstrated that averaging repeated TT measurements substantially improves measurement precision. The MDC95 decreased from 3.8 NRS points for a single measurement to 2.2 points when three measurements were averaged. This finding is most directly relevant to individual-level interpretation. When TT is used to evaluate change in a single participant, an observed change must exceed measurement error before it can be interpreted as reflecting true change. A single TT assessment therefore has limited precision for determining whether an individual participant has improved, whereas averaging repeated assessments reduces measurement error and brings the MDC95 closer to commonly used thresholds for clinically important improvement in chronic pain of approximately 2 NRS points or 30%.^29^

TT measurement precision has also implications for group-level treatment comparisons. However, in RCTs between-group differences are interpreted at the level of mean change or model-estimated treatment effects, and the precision of these estimates depends on sample size, between-person variability, within-person variability, the expected effect size, and the statistical model. Therefore, MDC95 should not be used as a direct threshold for judging whether a group-level treatment effect is clinically or statistically meaningful. However, reducing random measurement error by averaging repeated TT assessments may improve the precision of the outcome measure and can be relevant for sample-size planning and interpretation of longitudinal treatment effects. Future trials using TT as an outcome should therefore base power calculations on the variability of the chosen TT summary score, for example the variance of the mean of repeated TT assessments, or variance of a single TT measurement. The value of this approach must however, be balanced against participant burden and the specific aims of the trial. Moreover, repeated TT assessments do not seem to bring advantage in all type of trials. In the present study, correlations between TT pain and recalled intercourse pain changed only minimally when additional TT repetitions were averaged, suggesting that a single TT assessment may be sufficient for cross-sectional associations, whereas repeated assessments appear more relevant when TT is used to evaluate change over time.

### Convergent validity with intercourse-related pain

TT pain scores demonstrated moderate correlations with recalled pain during last intercourse. The correlations between TT pain and recalled pain during last intercourse support only partial convergent validity and should not be interpreted as evidence that the two measures are interchangeable. TT pain and intercourse pain are conceptually related, but they differ in important ways. The TT is a standardized, nonsexual provocation test, whereas intercourse-related pain occurs in a sexual and relational context and may be influenced by arousal, lubrication, partner interaction, movement and friction, fear, avoidance, and willingness to continue despite pain. In addition, recalled intercourse pain may be affected by recall bias and by variation in the time elapsed since last intercourse.

In summary, TT pain should be considered a related but distinct outcome from intercourse-related pain. This interpretation is also consistent with the recently published COS for PVD treatment studies, which recommends both insertional pain in a nonsexual context and insertional pain in a sexual context as separate core outcomes.^9^

### Implications for future research

The findings highlight the importance of careful selection and operationalization of pain outcomes in PVD trials. Although, repeated TT assessments improve measurement precision they do not fully address the challenge posed by inherent within person pain variability. Moreover, repeated testing may increase participant burden and contribute to missing data. One potential strategy is to evaluate score variability after two repetitions and perform a third measurement only when differences exceed a predefined threshold (eg, ≥2 NRS points). However, such strategies may increase additional logistical complexity. Notably, the number of TT repetitions had only a negligible impact on the strength of the association between TT pain and intercourse-related pain. This suggests that a single TT measurement may be sufficient for cross-sectional association analyses, whereas longitudinal studies that aim to evaluate within-person change or individual-level clinical improvement should preferentially rely on the mean of repeated measurements. In group-level randomized comparisons, the mean of repeated TT assessments may improve outcome precision, but sample-size calculations and interpretation of treatment effects should be based on the expected between-group difference and the variance of the selected TT summary score.

Future trials should continue to combine standardized pain provocation measures with patient centered outcomes, in line with current core outcome recommendations.^9^ The optimal balance between measurement precision, feasibility, and participant burden remains an open question and will depend on specific study designs and research objectives.

### Strengths and limitations

Strengths of this study include a well characterized clinical sample, pragmatic inclusion criteria that enhance external validity, and a rigorous evaluation of TT measurement properties using repeated assessments and MDC95 estimation. Integration with findings from the prior feasibility study further strengthens the interpretability of the findings.

Several limitations should be acknowledged. The sample consisted predominantly of young, highly educated women recruited into a clinical trial in an urban Norwegian setting. This may limit generalizability to women with PVD who are older, less educated, from more diverse socioeconomic or cultural backgrounds, or who are not willing or able to participate in an intervention study.

Pain during intercourse was assessed by recall, which may be influenced by the time elapsed since last intercourse and by contextual factors. In addition, the TT protocol included only three repetitions within a short time frame. Therefore, we cannot determine whether the observed reliability, MDC95 values, or systematic reduction across repetitions would remain stable over longer intervals, such as weeks or months, or across different phases of the menstrual cycle or clinical course.

The TT measurement-property analyses and baseline primary-versus-secondary subgroup comparisons were not prespecified in the public trial registry (NCT04613713). However, the TT analyses were planned a priori for this baseline paper and were motivated by the earlier ProLoVe feasibility study, which had identified substantial short-term variability in repeated TT ratings.

Although repeated-measures GLM was used to provide a simple descriptive test of mean change across the three fixed TT repetitions, a mixed-effects framework would also have been appropriate and may be preferable in future studies with more missing data or more complex within-person modelling aims.

## Conclusion

Women enrolled in this pragmatic PVD trial demonstrated substantial symptom burden and clinical heterogeneity. Primary PVD was associated with higher TT pain than secondary PVD, while most other baseline characteristics were similar. Repeated TT assessments substantially improved measurement precision and should be considered when TT is used to evaluate within-person change or individual treatment response. For group-level treatment comparisons, repeated testing may improve precision of the outcome measure, but interpretation of trial effects should also account for sample size, variability, expected effect size, and the statistical model used.
